# Non-Destructive Eggshell Strength Assessment Using Hertz Contact Theory Part I: Theory and Applicability

**DOI:** 10.3390/foods12061189

**Published:** 2023-03-11

**Authors:** Bart De Ketelaere, Matthias Corion, Ines Adriaens, Paul Van Liedekerke, Wouter Saeys

**Affiliations:** 1 Division of Mechatronics, Biostatistics and Sensors (MeBioS)—Department of Biosystems, Katholieke Universiteit Leuven, Kasteelpark Arenberg, 30, 3001 Leuven, Belgium; 2 Division Animal and Human Health Engineering (A2H)—Department of Biosystems, Katholieke Universiteit Leuven, Kleinhoefstraat 4, 2440 Geel, Belgium; 3 BIOMATH—Department of Data Analysis and Mathematical Modelling, Ghent University, Coupure Links 653, 9000 Ghent, Belgium

**Keywords:** eggshell strength, Hertz contact theory, non-destructive measurement, Young’s Modulus, eggshell impact, quasi-static compression

## Abstract

In the egg industry, fast and highly reliable quality measurements are crucial. This study presents a novel method based on Hertz contact theory that allows for non-destructive determination of eggshell strength. The goal of the study was to evaluate the material strength (Young’s Modulus) and structural strength (stiffness) of eggshells. To this end, an experimental setup was constructed to measure the collision of an eggshell with a small steel ball, which was recorded using a laser vibrometer. The study analyzed a sample of 120 eggs and found a correlation of 0.85 between the traditional static stiffness measured during quasi-static compression tests and the stiffness obtained from the Hertz contact theory. The results show that Hertz contact theory is valid for small steel spheres impacting eggshells, while a sensitivity analysis indicated that the most important factor in determining the strength of the eggshell is the contact duration between the egg and the impactor. These results open up the possibility of grading eggs based on their shell strength in a non-destructive manner.

## 1. Introduction

Eggs produced commercially for the table egg market must meet strict standards to ensure that only those of the highest quality reach the consumer [1]. Unlike most commodities, the part of the egg that is actually eaten (the contents) is hidden from view by the presence of the eggshell. An intact, clean eggshell is, therefore, a prerequisite to ensure the quality and safety of the table egg. A uniform color, smoothness and absence of any visible defects are also considered to be important. Any egg which fails to meet these criteria is likely to be downgraded. Since this absence of defects in the shell, mainly cracks, is of utmost importance to the customer, much emphasis has been put on the assessment of shell strength so that eggs can resist impacts during handling and transport on their way from the henhouse to the consumer. The assessment of shell strength is not only important for breeders who wish to select hens for producing strong-shelled eggs [2], but also for routine quality control in egg sorting plants [3].

For many years, mechanical methods have been utilized to assess the physical quality of the shell, which is primarily determined by its strength and the existence of cracks [4,5]. Eggshell strength evaluation techniques can be generally categorized into direct and indirect methods [6]. The most commonly employed direct method is the compression fracture force measured during quasi-static compression [6,7,8]. This measure determines the material strength of the shell. Furthermore, puncture tests and impact tests are alternative methods. However, all these direct methods are destructive in nature. Another parameter related to the material strength itself is Young’s modulus. Young’s modulus is the ratio of stress, which has units of pressure, to strain, which is dimensionless; therefore, Young’s modulus itself has units of pressure. The determination of this Young’s modulus is not straightforward in the case of eggshells due to its particular dimensions. Furthermore, measurements are destructive and very time-consuming due to eggshell sample preparation and standardization [9].

Indirect techniques, including both destructive and non-destructive, measure a parameter that correlates with the actual ability of eggshells to resist breakage in practice [6]. Eggshell thickness is commonly used as an indirect measure for the eggshell’s strength. Alternatively, the strength of the egg can also be indirectly determined by calculating the weight percentage that is attributed to its shell. A popular indirect and non-destructive technique is the quasi-static compression of the egg between two parallel plates. By calculating the slope of the force–deformation curve, the static stiffness of the egg (*K_stat_*) can be estimated. Additionally, the non-destructive deformation refers to the degree of bending or deflection of an eggshell under an applied force. This structural property of the egg can be utilized to estimate the force necessary to fracture the shell [6]. Although indirect methods are used to measure eggshell strength based on the assumption that they are correlated with direct values, the moderate observed correlations among those parameters suggest the involvement of several other factors in shell fracture. Moreover, the quasi-static approach of the above-mentioned non-destructive shell strength assessment methods requires compression at low speeds, prohibiting an inline use.

While some of the techniques mentioned above can be categorized as ‘non-destructive’, their time-consuming nature limits the evaluation to small samples of large shipments. This resulted in the development of new mechanical sensors that are both reliable and fast, with some already available on the commercial market [4]. For instance, Moayeri presented a set-up where a small impactor is used to excite the eggshell [10]. Here, the number and amplitude of rebounds of the impactor are used as measures for the local mechanical eggshell integrity. A surface that is locally intact allows for multiple elastic rebounds with high amplitude, whereas a crack in the adjacent shell area seriously impairs the elasticity, leading to significantly damped rebounds. In contrast, Coucke proposed an approach where the response of the egg upon impact was evaluated rather than analyzing the impactor’s behavior following excitation [11]. When an egg is subjected to a non-destructive impact excitation, an oscillation response will be produced by the shell. Coucke [11] and De Ketelaere [12] observed that eggs with damaged shells exhibit a greater number of resonant peaks than undamaged ones. Moreover, they found that for intact eggs, the impulse response was nearly identical at all points on the equator. In contrast, damaged shells displayed different responses at distinct equatorial locations. This research inspired several other researchers to build accurate crack detection algorithms based on the vibration of the egg [13,14,15,16,17,18].

Besides using vibration analysis for crack detection, Coucke [11], Coucke et al. [19] and De Ketelaere et al. [12,20] used the technique for estimating the shell strength of intact eggs. Therefore, Coucke [11] performed dynamic impact measurements and modelled the egg as a mass–spring system. Here, he defined a novel eggshell strength parameter named dynamic stiffness, *K_dyn_*. Coucke [11] and Coucke et al. [19] reported a correlation of 0.71 between static and dynamic stiffness, while De Ketelaere et al. [12,20] extended the mass–spring model to a mass–spring–damper model and showed that damping of the vibration provides additional information. Later, Sun et al. [21] confirmed that an acoustic impulse response approach allowed for deriving shell strength information. Furthermore, the heritability of dynamic stiffness has been demonstrated to be moderate to high by Dunn et al. [22]. This indicates that breeding companies can incorporate this new parameter into their selection schemes for improving shell strength. However, it remains to be proven whether genetic selection based on dynamic stiffness adds to reducing the probability of an egg breaking in practice, which is the final goal for any selection program for shell strength. Nedomova et al. [23] proposed an experimental method for the evaluation of the eggshell’s mechanical characteristics under impact loading. By gradually increasing the rod impact velocity, the rupture force of the eggshell was determined. Sun et al. [21] combined frequency analysis and chemometrics to derive strength information with moderate accuracy.

In summary, shell strength is a complex matter, and it is still unclear whether classical or newly developed shell strength indices, such as the dynamic stiffness, although promising, have sufficient predictive power with respect to breakage in practice. Given the economic importance of shell strength, there remains a demand for more accurate and/or faster indicators for shell strength.

Therefore, the goal of this study was to provide a sound basis for the development of a reliable and fast method for assessing shell strength using impact measurements. It distinguishes itself from other research in the following aspects:

- Hertz contact theory is used to describe the actual impact on the egg by a small sphere. This is substantially different from the Moayeri [10] application, in which rebounds are counted as a measure of local shell stiffness;
- The research concerning the vibration of the egg after impact by a small sphere focuses on the impulse response of the egg itself.

The benefit of such a method is twofold: (1) it could open the door towards a new, fast measurement set-up, and (2) it could add information on the probability of shell breakage in practice.

## 2. Materials and Methods

### 2.1. Experimental Set-Up

**Figure 1:**
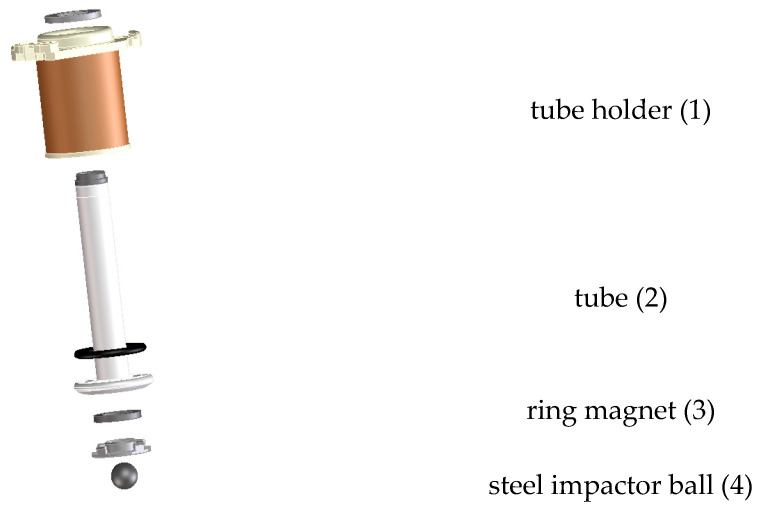
Impactor consisting of a plastic tube with a ring magnet at its lower end, in which magnetic field a steel impactor ball is positioned. Picture provided by Moba BV.

For impacting the eggs, a commercial impactor included in a Moba (Moba BV, Barneveld, The Netherlands) crack detection system was used (Figure 1). It consists of a short plastic tube (2) that is equipped with a ring magnet (3) at its end. In this magnetic field, a small steel ball (4) is placed. The steel ball has a radius of 4.5 mm and weighs 3 g. The tube can move within a tube holder (1). Typically, the tube holder is fixed above the egg, and the tube is then released so that it falls under gravity, and the impactor ball that is floating in the magnetic field touches the egg. The ball will rebound several times on the egg surface before coming to rest on the egg surface.

As the impactor ball can only move in one direction (the direction of the tube), a laser vibrometer (OCV 3001, Polytec GmbH, Waldbronn, Germany) could be used for accurate impact measurements. The laser was positioned above the plastic tube, with the laser beam pointing downward through the tube to be reflected by the ball at the end of the tube. The laser signal was digitized using a National Instruments^®^ (Austin, TX, USA) data acquisition board connected to a personal computer. Preliminary experiments were executed to determine a suitable sampling rate to analyze the impact behavior. It was fixed at 50 kHz throughout all experiments. Digitized signals were further analyzed using Matlab^®^ software (Matlab version R2021a, The Mathworks, Inc, Natick, MA, USA).

The eggs that were impacted were supported by two diabolo-shaped rollers, as is classically done on commercial grading machines, while the probe was directed to the equator of the egg where the actual impact occurs.

### 2.2. Egg Samples and Egg Characteristics

A total of 120 fresh, brown-shelled consumption eggs taken from a local warehouse were used to investigate the relation between the static stiffness, *K_stat_*, measured under quasi-static compression (see below) and the Hertz stiffness, *K_H_*, determined in the experimental set-up described in the above section. The egg mass varied between 52 and 76 g; static stiffness varied between 134 and 211 kN/m. Each egg was measured once using the above-described setup.

To measure static stiffness (*K_stat_*), eggs were horizontally placed between two flat parallel steel plates and compressed at a rate of 10 mm/minute using a universal tensile and compression test machine (UTS Testsysteme GmBh., Denkendorf, Germany). The resolution of the force sensor was 0.001 N, and a maximum force of 10 N was exerted. Throughout the test, both force and deformation were recorded, and static stiffness was calculated by determining the slope of the force-deformation curve between 0.98 and 10 N. The measurement was repeated on three equidistant places at the equator of the egg. The average value of the three measurements was used further in the statistical analysis. The egg curvature was determined by taking the egg diameter at the equator (being the impact location, see above), measured by sliding calipers with an accuracy of 1 mm.

### 2.3. Hertz Contact Theory

Consider two quasi-elastic spherical bodies of mass *m*_1_ and *m*_2_. (i.e., the impactor ball and the egg). The motion of the bodies towards each other can be described by the normal velocities of their centers of mass, *v_n_*_1_ and *v_n_*_2_. During collision, the two bodies will each be subjected to a normal force *N* acting at the surface of contact, and the centers of the spheres approach each other by a normal deflection *δ_n_*, which can be regarded as a virtual overlap distance between the two spheres. Their relative normal velocity, *v_n_* (m/s), is given by:

(1) $$v_{n}=v_{n1}-v_{n2}=\frac{{d\delta }_{n}}{dt}$$

The force balance at collision can be determined from the rate of change of the momentum for each particle as:

(2) $$m_{1}\frac{{dv}_{n1}}{dt}=-N,\;m_{2}\frac{{dv}_{n2}}{dt}=N$$

By solving the above simultaneous Equation (2) and using (1), the following equation can easily be derived:

(3) $$\mu \frac{d^{2}\delta_{n}}{{dt}^{2}}=\mu \frac{d}{dt}\left(v_{n2}-v_{n1}\right)=N$$

where the effective mass of the system, *µ* (kg), is given by:

(4) $$\mu =\frac{m_{1}m_{2}}{m_{1}{+m}_{2}}$$

The most accurate expression for the normal force in the case of two elastic spheres in contact is according to the Hertz theory [24], which prescribes the following:

(5) $$N=-K_{H}\delta_{n}^{3/2}$$

where the Hertz stiffness constant, *K_H_* (N/m^3/2^), is given by:

(6) $$K_{H}=\frac{4}{3}{\tilde{R}}^{1/2}\tilde{E}$$

Here, $\tilde{R}$ and $\tilde{E}$ are defined, respectively, as the effective radius and the effective Young’s modulus of the system, and they are given by:

(7) $$\frac{1}{\tilde{R}}=\frac{1}{R_{1}}+\frac{1}{R_{2}}$$

and

(8) $$\frac{1}{\tilde{E}}=\frac{1-\nu_{1}^{2}}{E_{1}}+\frac{1-\nu_{2}^{2}}{E_{2}}$$

where *R*_1_ and *R*_2_ (m) are the radii of curvature for the two bodies, *E*_1_ and *E*_2_ are the Young’s moduli (N/m²), and *ν*_1_ and *ν*_2_ are Poisson ratios of the two colliding bodies. In principle, in a normal collision between two bodies, dissipative forces will be present, as well (see [25]). Here, we will neglect these because of the quasi-elastic nature of the two bodies.

By substituting the Hertz normal force relation above, (5), in (3), we can write the dynamic equation for the collision of two spheres as:

(9) $$\mu \frac{d^{2}\delta_{n}}{{dt}^{2}}+K_{H}\delta_{n}^{3/2}=0$$

Using the conservation of the total (kinetic and elastic) energy of the system, one also has that:

(10) $$\frac{1}{2}\left(v_{n0}^{2}-{\left(\frac{{d\delta }_{n}}{dt}\right)}^{2}\right)=\frac{2}{5}\frac{K_{H}}{\mu }\delta_{n}^{5/2}$$

where *v_n0_* is the initial relative impact velocity. By setting *dδ_n_/dt* to zero, we can derive an expression for the maximum normal deflection *δ_n_^max^*:

(11) $$\delta_{n}^{max}={\left(\frac{{5\mu v}_{n0}^{2}}{{4K}_{H}}\right)}^{2/5}$$

Alternatively, the Hertz theory provides a solution to the contact duration, *τ,* between the two spheres as a function of the impact velocity, *v_n0_*, and the maximal deformation, *δ_n_^max^* [26]:

(12) $$\tau =2\frac{\delta_{max}}{v_{n0}}\int_{0}^{1}\frac{dv}{\sqrt{{1-v}^{5/2}}}≅2.94\frac{\delta_{max}}{v_{n0}}$$

Inserting now Equation (11) into Equation (12) leads to an expression for the contact duration, *τ*, as a function of the impact velocity, the effective mass and the Hertz stiffness constant, *K_H_*:

(13) $$\tau =c{\left(\frac{\mu }{K_{H}}\right)}^{2/5}v_{n0}^{-1/5}$$

with *c* denoting a constant equal to 3.2145. The general equations derived above will serve as a basis for the egg–impactor case considered in this research. Note that a small correction should be considered in Equation (5) if one body is only approximately spherical, which is particularly the case for eggs. Here, this correction will be neglected because the radius of the other body (steel ball) is significantly smaller. For a more detailed analysis of this topic, the reader is referred to [27].

### 2.4. Calculation of the Shell’s Young’s Modulus

Solving the Hertz equations for the Young’s modulus of the egg can be achieved in different ways. The most straightforward way is to start from Equations (6)–(8) and substitute the values into Equation (13). This leads to the following relation, in which the Young’s modulus of the egg, *E*_2_, is expressed as a function of the impact speed, *v_n_*_0_, the impact duration, *τ*, and known parameters.

(14) $$E_{2}=\frac{1}{2}\left(\frac{\left({-18c}^{3}{\mu +18c}^{3}{\mu v}_{1}^{2}{+24E}_{1}\tau^{2}\sqrt{{cR\tau v}_{n0}}\right)\left(v_{2}+1\right)\left(v_{2}\;-\;1\right)c^{2}E_{1}\mu }{{16RE}_{1}^{2}\tau^{5}v_{n0}\;{-\;9c}^{5}\mu^{2}{+18c}^{5}v_{1}^{2}\mu^{2}\;{-\;9c}^{5}v_{1}^{4}\mu^{2}}\right)$$

Alternatively, *E*_2_ could be expressed in terms of the maximal deformation and the impact speed, but these calculations are less straightforward. When inspecting Equation (14), there are three main influencing factors: egg radius (and, thus, egg mass), impact speed and contact duration.

A sensitivity analysis was performed to investigate the effect of small changes in those three main parameters on the estimation of the *E*_2_. The egg radius was altered between 0.015 and 0.03 m, whereas the impact speed was altered between 0.1 m/s and 0.6 m/s, all being realistic values for egg impact.

### 2.5. Data Analysis

The results section presents data as measured during the experiments described above. All data visualizations and analyses were performed using Matlab^®^ software (Matlab version R2021a, The Mathworks, Inc, Natick, MA, USA). For relating the different strength measurements, a simple linear regression was performed, and Pearson correlation coefficients were reported.

## 3. Results & Discussion

### 3.1. Impact Data Description

**Figure 2:**
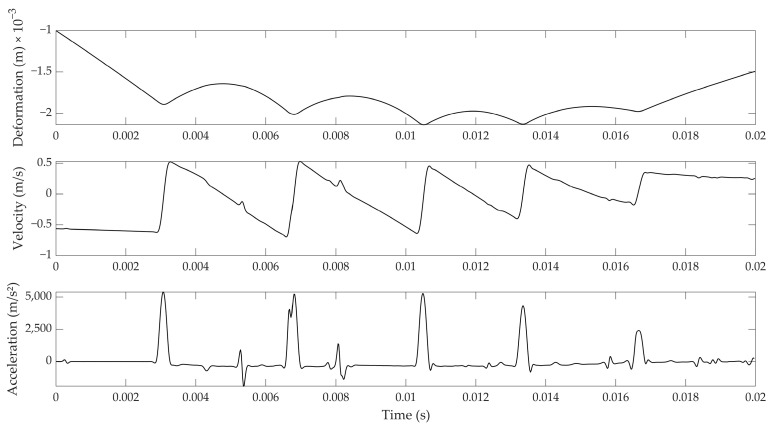
Impact on an intact egg by a small steel ball: Velocity data retrieved from sampling the laser vibrometer at 50 kHz (**middle**); Deformation (m) signal obtained through integration (**top**); and Acceleration signal (m/s²) obtained through derivation (**bottom**).

When the tube depicted in Figure 1 was dropped onto the egg surface, a typical signal given in Figure 2 was obtained. The top figure shows the five consecutive rebounds of the impactor ball from the egg surface. This deformation signal was obtained by integrating the velocity signal measured by the laser vibrometer, which is shown in the middle figure. The acceleration of the ball shown in the bottom figure was obtained as the time derivative of the velocity signal.

**Figure 3:**
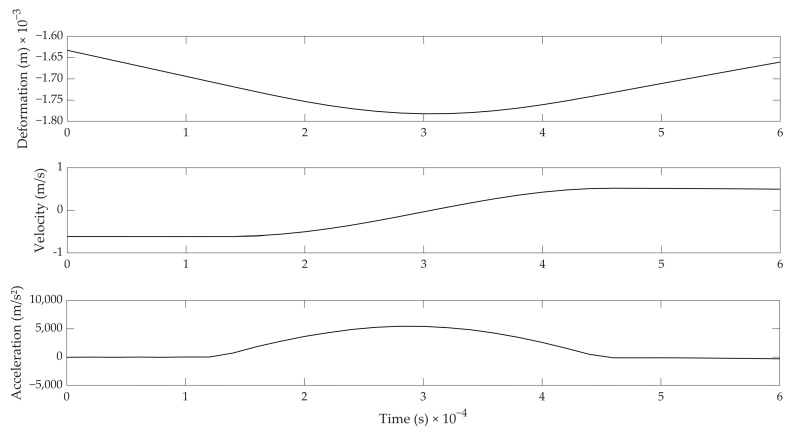
Zoomed view of the first impact on the egg with the steel ball. (**Top**): Deformation (m); (**middle**): velocity (m/s); and (**bottom**): acceleration (m/s²).

For investigating the general applicability of Hertz contact theory, only the data related to the impact loading and unloading were used. Figure 3 gives a zoomed view of the first impact that was shown in Figure 2. As can be seen from the acceleration signal, the typical contact duration between the egg and the ball was approximately 0.3 milliseconds, while the maximal acceleration reached about 5000 m/s². The deformation of the eggshell is less easy to derive from the Figure, but was approximately 35 µm on average—or about 10% of the eggshell thickness.

### 3.2. Applicability of the Hertz Equations

**Figure 4:**
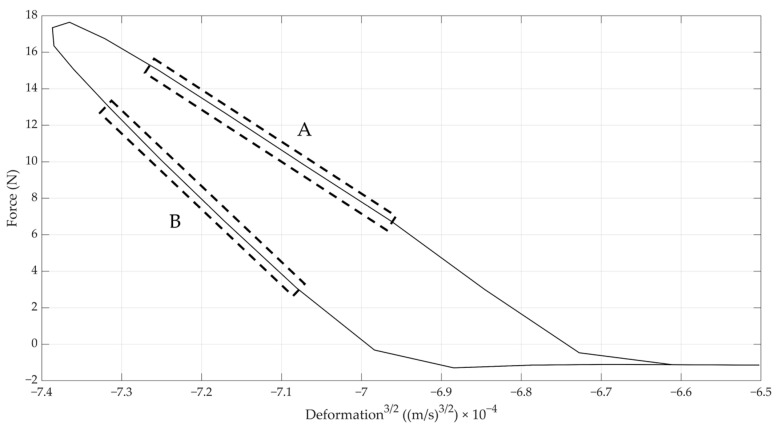
Loading (**A**) and unloading (**B**) of the egg by the steel ball. Linear regions as expected based on Hertz theory are highlighted.

Three different checks were performed for judging the applicability of the Hertz contact theory and, thus, the further derivation of shell strength based on it. First, the applicability of Hertz theory was verified by plotting the experimental relation between the force exerted by the impactor on the egg versus the displacement to the power 3/2, and checking whether the relation was linear, as described by Equation (5). The force, *N*, was calculated by taking the product of the mass of the ball (i.e., 3 g) and the measured acceleration of the ball (see Figure 2 and Figure 3). This relation is illustrated in Figure 4 for the loading and unloading of the egg. It can readily be seen that this relation is quasi-linear, proving Hertz theory was valid.

**Figure 5:**
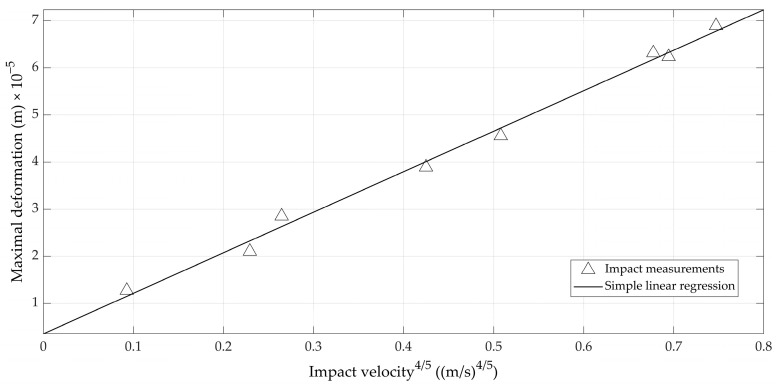
Relation between the impact velocity and the maximal deformation of the eggshell.

A second check of the applicability of the Hertz contact theory was provided by plotting the experimental relation between the impact speed on the egg to the power (4/5) versus the maximal deformation, and checking whether it was linear, as described by Equation (11). For the relationship depicted in Figure, consecutive rebounds of the impactor ball on an example egg were used. It can readily be seen from Figure 5 that the relation indeed was linear (*r* = 0.997).

**Figure 6:**
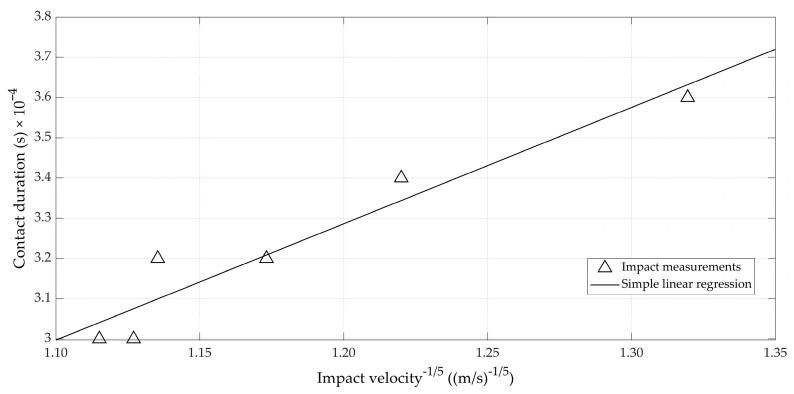
Relation between the impact velocity and the contact duration of the collision.

A last check concerned the relation presented in Equation (13) between the contact duration and the impact velocity to the power (−1/5). In order to do so, consecutive hits with the impacting probe for a given egg were considered, in analogy to the measurements that were shown in Figure 2. The contact duration was derived from the acceleration signal (Figure 3, bottom). The start of the impact was defined as the moment at which the acceleration exceeded 50 m/s², and the end of the impact was similarly defined as the moment at which the acceleration went back below this limit value of 50 m/s². A total of six impacts were retrieved for constructing Figure 6. Although there were only a limited number of impacts for an individual egg using the set-up, it can be seen that linearity as prescribed by Equation (13) was not contradicted (*r* = 0.959).

### 3.3. Sensitivity Analysis for Young’s Modulus

**Figure 7:**
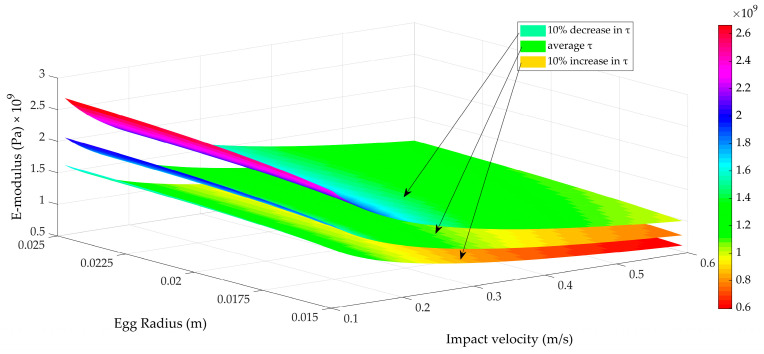
Sensitivity analysis showing the influence of the impact velocity (x-axis), egg radius (y-axis) and the contact duration (given as three separate surfaces) on Young’s (or E) modulus (z-axis).

A sensitivity analysis was performed to investigate the influence of the egg radius and the impact speed. Three distinct values were used for the impact duration to cover a broad range of eggshell strengths. For each value chosen, a different surface is plotted in Figure 7. Values used for the contact duration were 0.34 ms ± 10%.

Two main observations can be made from Figure 7. The absolute values of the estimated Young’s moduli obtained are well below the values reported in literature, as most authors obtained values around 30 GPa (summarized in [9]), while our estimates are around 2 GPa. This may be attributed to the substantial deformation resulting from the impact (about 30 to 70 µm, Figure 3 and Figure 5) with respect to the shell thickness (350 µm on average). It is, therefore, hypothesized that not only the shell was locally deforming, but the whole egg as a structure deformed during the impact. The E-moduli calculated from the impact experiments are, thus, related to the structural strength of the egg (combination of the material strength and geometry, shell thickness, etc.) rather than to the eggshell material strength alone.

A second observation relates to the main influencing parameters. As expected, the egg curvature did not play a major role: in Hertz contact theory, the smallest sphere mainly determines Equations (4) and (7). The other two parameters, impact velocity and contact duration, are both of major importance. The impact velocity was most important when its value was low (<0.2 m/s). For higher values, the influence of impact velocity became less important. Altering the impact duration by 10% seemed to have a repercussion for all impact velocities considered. With the above observations in mind, it seemed plausible to determine the shell strength based on an accurate determination of the contact duration, augmented by an impact velocity measurement when impact velocities were low.

### 3.4. Calculation of Hertz Stiffness and Its Relation to Static Stiffness

As highlighted in the introduction, the stiffness of the egg is an important parameter describing its quality, but the quasi-static measurement is time-consuming. Therefore, it is investigated in this section whether it could be assessed using the simple impact test used in this research.

The starting point for the calculations is Equation (13) relating the impact speed and the impact duration, in combination with the mass *µ* of the system to the Hertz stiffness *K_H_*. After solving for *K_H_*, this equation reads:

(15) $$K_{H\;}={\left(\frac{c}{\tau }\right)}^{5/2}v_{n0}^{-1/2}\mu$$

**Figure 8:**
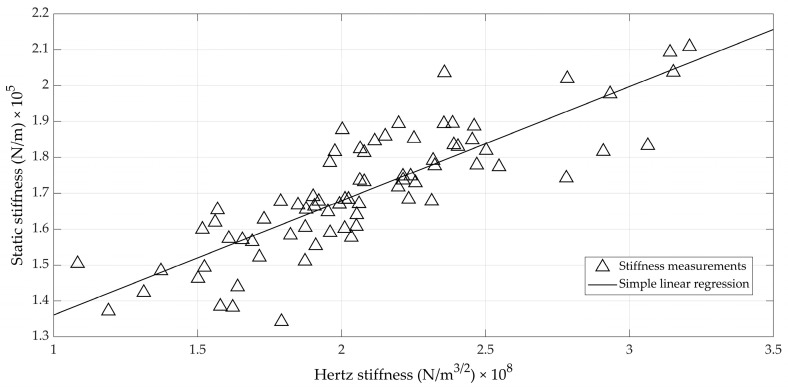
Scatterplot of static stiffness measured during quasi-static compression using a Universal Test Machine as a function of the Hertz stiffness measured in the experimental set-up.

A scatterplot of the relation between the static stiffness measured using the Universal Testing Machine and the Hertz stiffness measured on the experimental set-up is shown in Figure 8. It can readily be seen that the relation between both is moderate (*r* = 0.84). As can be derived from Equation (15), the impact duration is mainly responsible for the observed correlation.

**Figure 9:**
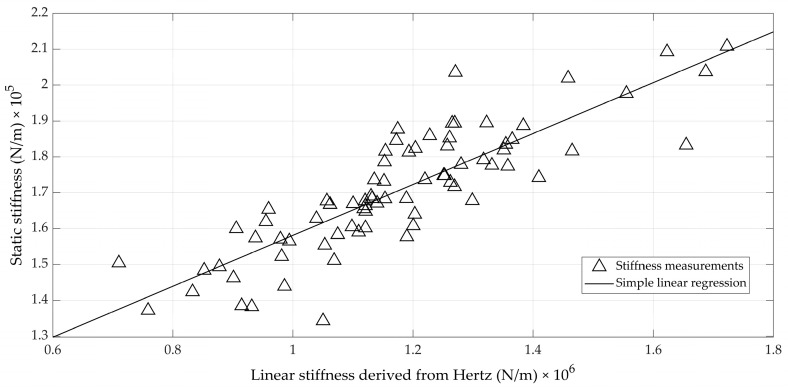
Scatterplot of the static stiffness measured during quasi-static compression using a Universal Test Machine as a function of the linear stiffness derived from Hertz theory.

Alternatively, Figure 9 shows the relation between the classical static stiffness and a linear stiffness that is deduced from taking the ratio between the maximal force and the maximal deformation during impact. This ratio between maximal force and maximal deformation, thus, has the same units as the classical *K_stat_*. The maximal deformation is calculated using Equation (11). The maximal force is determined by inserting the value for the maximal deformation of Equation (11) into Equation (5). It has a similar linear correlation with *K_stat_* (*r* = 0.85).

It can be observed from Figure 9 that the nominal values of both stiffnesses were quite different: the values obtained from the impact measurements and Hertz contact theory were about eight times higher than those obtained from the quasi-static measurements. It is obvious that the impact (compression) speed differed greatly between both approaches, providing the main explanation. The impact speed during the Hertz measurements was around 0.5 m/s (see, for instance, Figure 2), whereas the compression speed during the UTS measurements was only 10 mm/min. On the other hand, typical deformations for quasi-static compression tests up to 10 N, as used here (typically around 60 µm), were about double when compared to the impact test (35 µm on average), although the maximal forces exerted in the impact test were higher (20 N on average; see, for instance, Figure 4). In summary, it can be stated that the linear Hertz stiffness given in Figure 9 related well to the classical static stiffness, but could be labelled “dynamic” and, thus, provided different nominal values.

The correlations between the classical stiffness, *K_stat_*, and the newly defined stiffness (*K_H_*, or its linear version from Figure 9) were moderate to high (correlation coefficient around 0.85) and were typical for this kind of measurement on biological material. In their study of the vibrations of impacted eggs, Coucke [11] and De Ketelaere et al. [4,20] reported correlations between the so-called dynamic stiffness, *K_dyn_*, and *K_stat_*, ranging from 0.65 to 0.85. This might not be surprising, since the intra-egg variability in stiffness (due to, for instance, uneven distribution of the shell thickness around the equator) augmented by the measurement error during compression typically leads to coefficients of variation of about 5%. Indeed, in this study, the average standard deviation of the three measurements of *K_stat_* for each egg was 5640 N/m. This value can serve as a benchmark for determining the minimum level of accuracy that any technique under comparison must surpass. The error standard deviation of the linear model from Figure 9 to predict *K_stat_* was 9390 N/m, about 50% higher than the standard deviation of the reference measurement.

## 4. Conclusions

This study showed that the classical Hertz contact theory accurately describes the case of impacting whole-shelled eggs with a small metal sphere. It was shown that the contact duration between the egg and the impactor is an important indicator for the egg strength. The impact velocity only has a major influence at low values (below 0.2 m/s). Given the large ratio between the egg and the impactor radius, the egg radius and mass influence is negligible.

The eggshell Young’s moduli estimated from the impact measurements were considerably lower than the values reported by other researchers. It was hypothesized that this can be attributed to the representation of an egg as a homogenous sphere, which ignores the real structure involving a thin shell surrounding the inner content that has virtually no structural strength.

The estimates for the Hertz stiffness correlated well with the static stiffness, the most widely used parameter for shell strength. Adopting this approach holds the potential to deliver non-destructive and rapid assessment of egg strength and to offer valuable insights into the probability of egg breakage in practice.

## Data Availability

The data presented in this study are available on request from the corresponding author. The data are not publicly available for proprietary reasons.
